# Configurational and predictive mechanisms of performance anxiety in female collegiate athletes: the synergistic roles of evaluative pressure, self-efficacy, and mental toughness

**DOI:** 10.3389/fpsyg.2026.1898041

**Published:** 2026-09-16

**Authors:** Yuhang Lei, Li Cai

**Affiliations:** School of Sports Science, Nanjing Normal University, Nanjing, Jiangsu, China

**Keywords:** fear of negative evaluation, fuzzy-set qualitative comparative analysis (fsQCA), mental toughness, partial least squares structural equation modeling (PLS-SEM), performance anxiety, self-efficacy

## Abstract

**Background:**

Chinese University Basketball Association League (CUBAL) Division II female athletes face significant performance anxiety. Moving beyond traditional linear assumptions, this study integrates self-presentation theory and the positive psychological capital framework to investigate how external evaluative pressure (fear of negative evaluation), internal resources (self-efficacy, mental toughness), and competitive experience synergistically predict performance anxiety.

**Methods:**

A dual-analytical design was employed with 143 female athletes. Partial least squares structural equation modeling (PLS-SEM) tested linear, mediating, and moderating effects. Concurrently, fuzzy-set qualitative comparative analysis (fsQCA) evaluated condition necessity and asymmetric sufficient configurational paths.

**Results:**

PLS-SEM demonstrated that fear of negative evaluation positively predicted performance anxiety and negatively predicted self-efficacy. Self-efficacy partially mediated this relationship, whereas mental toughness directly mitigated anxiety and moderated the self-efficacy–anxiety linkage. Necessity analysis indicated no single condition was necessary for high anxiety. fsQCA identified two equifinal paths to high performance anxiety and one path to non-high anxiety, verifying significant configurational asymmetry.

**Conclusion:**

Performance anxiety results from the complex, asymmetric synergy of external pressure, resource deficiencies, and experience states. Positive psychological capital effectively compensates for experiential disadvantages. Conversely, extensive competitive experience coupled with deficient psychological resources exacerbates anxiety risk, challenging the linear assumption of experience as a universally protective factor. These findings provide empirical foundations for targeted psychological interventions in resource-constrained competitive environments.

## 1 Introduction

Performance anxiety is a key antecedent variable associated with athletes’ performance and mental health in competitive sports environments (Mellalieu et al., 2009; Rice et al., 2016). Existing studies have predominantly focused on professional elite or CUBAL Division I athletes, whereas empirical investigations into the general college student-athlete population remain relatively limited. In the Chinese University Basketball Association League (CUBAL) Division II system, the participants are primarily ordinary full-time college students. Compared to professional or high-level athletes, this cohort faces a systemic lack of professional medical and psychological support systems during daily training and competition (Condello et al., 2019). Meanwhile, the need to meet the same academic assessment criteria as ordinary students means that cyclical, high-intensity competition preparation objectively exacerbates their “academic-training” role conflicts (Aquilina, 2013). Furthermore, relevant empirical studies indicate that female athletes exhibit specific cognitive and emotional reaction characteristics when coping with external competitive pressure and social evaluation (Kristjánsdóttir et al., 2018). The convergence of the aforementioned structural resource constraints and specific group characteristics constitutes the practical rationale for examining the competitive psychological profile of this population.

Regarding the mechanisms underlying performance anxiety, self-presentation theory provides a framework for explaining the internalization of external environmental stress. This theory posits that when individuals anticipate an inability to convey a positive image to key audiences such as spectators, coaches, or peers, fear of negative evaluation arises (Leary, 1992). In competitive sports contexts, such persistent apprehension about external negative social evaluation has been proven to be a core proximal stressor linked to performance anxiety (Mesagno et al., 2012). When coping with such external evaluative pressure, an individual’s internal psychological capital resources play a pivotal role in maintaining psychological stability. Among these, self-efficacy and mental toughness are regarded as crucial positive psychological resources for buffering against external threats (Gucciardi et al., 2015; Poulus et al., 2024; Robazza et al., 2025). However, current empirical frameworks in sports psychology predominantly focus on the linear main effects of a single external stressor or internal trait on competitive anxiety (Woodman and Hardy, 2003; Hanton et al., 2008), heavily relying on symmetric variance analysis techniques such as structural equation modeling (SEM) or multiple regression (Sarstedt et al., 2022; Kline, 2023).

Although existing studies have established a significant theoretical foundation, there remain theoretical and methodological gaps in the exploration of complex competitive contexts that warrant further investigation. The current literature has not adequately integrated the background of role conflicts and support resource constraints faced by female college basketball players, lacking in-depth empirical evidence on the synergistic mechanisms of external evaluative pressure and internal psychological resources within specific constrained situations. Furthermore, previous studies have generally held a linear assumption regarding “competitive experience,” tending to view it independently as a positive factor that suppresses anxiety, with little consideration given to the adverse expectancy burdens during the accumulation of experience. Methodologically, traditional symmetric models have inherent limitations in evaluating the concurrent effects of multiple variables: such methods fail to address the issue of configurational asymmetry—that is, the combinations of conditions associated with high anxiety are not merely the inverse of those associated with low anxiety (Woodside, 2014). Moreover, traditional models assume that each variable independently competes to explain the variance, neglecting the configurational interaction effects among multidimensional psychological characteristics and objective experience variables, which is insufficient to accurately identify the complex sufficient configurations underlying emotional states (Fiss, 2011; Pappas and Woodside, 2021). Consequently, what remains unknown is how external evaluative pressure, internal psychological resources, and competitive experience interact in complex, asymmetric configurations to predict performance anxiety (Woodside, 2014). Specifically, within the resource-constrained context of Division II female athletes, the synergistic combinations of conditions that lead to, or avert, high performance anxiety have yet to be identified. The scientific novelty of this study lies in shifting the paradigm from a purely linear perspective to a configurational one, explicitly addressing this unknown by mapping the asymmetric pathways of performance anxiety.

To overcome the existing theoretical and methodological limitations, this study adopts a dual-analytical design using partial least squares structural equation modeling (PLS-SEM) and fuzzy-set qualitative comparative analysis (fsQCA). Based on self-presentation theory and the positive psychological capital framework, this study explores the synergistic influence mechanisms of competitive experience, fear of negative evaluation, self-efficacy, and mental toughness on the performance anxiety of Division II athletes. Specifically, PLS-SEM is employed to test the linear main effects, as well as the mediation and moderation paths among the variables; simultaneously, fsQCA is utilized to conduct necessity analysis and reveal the asymmetric sufficient configurational paths leading to high (and non-high) performance anxiety. The theoretical contributions of this study are as follows: (1) integrating external evaluative pressure and internal psychological capital into the same model, thereby broadening the nomological network of antecedents for performance anxiety in the Division II cohort; (2) verifying the complex nonlinear synergistic effects of competitive experience and psychological traits in inducing or averting anxiety; and (3) utilizing a configurational perspective to elucidate the multiple pathways of anxiety among female college student-athletes under specific resource constraints, thus providing a scientific basis for precise and resource-efficient psychological intervention practices in grassroots teams.

Self-presentation theory posits that individuals are inherently motivated to control the impressions others form of them during social interactions. In competitive sports contexts, when athletes anticipate an inability to present a positive image to spectators, coaches, or teammates, or when they worry about exposing competence deficits, a heightened fear of negative evaluation is induced (Schlenker, 1980). This anticipatory apprehension regarding negative social evaluation constitutes a core proximal stressor that is associated with competitive anxiety (Mesagno et al., 2012). Concurrently, positive psychological capital, which encompasses core elements such as self-efficacy and mental toughness, has been widely demonstrated to be a crucial internal resource for buffering against external stressors (Luthans et al., 2015). Self-efficacy denotes an individual’s belief in their capability to execute specific competitive tasks, whereas mental toughness represents a psychological trait that enables individuals to maintain high performance levels when facing pressure and adversity (Gucciardi et al., 2015). In the objectively resource-constrained environment of CUBAL Division II, where external professional psychological interventions are scarce, athletes’ capacity to withstand external evaluative pressure relies heavily on the availability and mobilization of their internal positive psychological capital.

This study introduces cognitive appraisal theory to elucidate the theoretical predictive pathways between evaluative pressure and internal psychological resources. This theory maintains that external stressors must undergo an individual’s cognitive appraisal resulting in specific emotional and behavioral reactions (Lazarus and Folkman, 1984). Following the logic of this framework, fear of negative evaluation, as an environmentally imposed social stressor, first disrupts an individual’s self-competence appraisal system, thereby undermining their self-efficacy beliefs (Bandura, 1997). Consequent to the continuous depletion of cognitive resources and a decline in self-confidence, individuals become incapable of effectively coping with immediate competitive challenges, which ultimately culminates in performance anxiety. Therefore, this study contends that fear of negative evaluation not only predicts performance anxiety but also explains psychological exhaustion and anxiety via the mediating mechanism of compromised self-efficacy.

In sports science literature, “competitive experience” is conventionally viewed as an inherent buffer against stress. However, as competitive experience accumulates, the external expectations (e.g., assuming a core role in the team) and internal pressure faced by athletes increase correspondingly (Mellalieu et al., 2009). When senior athletes’ internal psychological capital (e.g., self-efficacy and mental toughness) is insufficient to manage the high expectancy load brought by experience, experience transforms into a conditional variable that may exacerbate anxiety. Furthermore, the internal elements of psychological capital do not operate in isolation: a high level of mental toughness can serve as a self-regulatory resource, altering an individual’s threat appraisal of stressful situations, thereby exerting a buffering effect on the predictive relationship between self-efficacy and performance anxiety (Crust, 2008).

Based on the aforementioned theoretical deductions, elevated fear of negative evaluation substantially depletes athletes’ cognitive resources, diverting their attention from task execution to social evaluation threats, which predicts performance anxiety (Mesagno et al., 2012). Concurrently, this fear is linked to self-doubt, significantly reducing athletes’ self-efficacy regarding the achievement of competition goals (Feltz et al., 2008). In turn, diminished self-efficacy weakens individual coping capacities, further amplifying performance uncertainty and anxiety. Consequently, we propose the following hypotheses:

*H1*: Fear of negative evaluation significantly and positively predicts performance anxiety.

*H2*: Fear of negative evaluation significantly and negatively predicts self-efficacy.

*H3*: Self-efficacy significantly and negatively predicts performance anxiety.

According to the transmission logic of cognitive appraisal theory, this study asserts that fear of negative evaluation, as an external environmental stressor, can indirectly predict performance anxiety by undermining an individual’s self-efficacy. Self-efficacy constitutes a critical mediating variable along the pathway from “fear” to “anxiety.” Consequently, we propose the following hypothesis:

*H4*: Self-efficacy plays a partial mediating role in the relationship between fear of negative evaluation and performance anxiety.

In accordance with the buffering hypothesis of positive psychological capital, mental toughness cannot only directly suppress anxiety as an independent resource but also moderate the pathways of other variables during complex stress processes (Gucciardi et al., 2015). Athletes with high mental toughness are capable of maintaining a strong goal focus, thereby mitigating the negative impact of self-efficacy fluctuations on anxiety. Consequently, we propose the following hypotheses:

*H5*: Mental toughness significantly and negatively predicts performance anxiety.

*H6*: Mental toughness significantly moderates the predictive relationship between self-efficacy and performance anxiety.

Based on the preceding theoretical deductions and analyses, the core research hypotheses (H1–H6) of this study have been formulated. Figure 1 illustrates the conceptual framework model derived from the aforementioned analysis, systematically elucidating the structural and predictive relationships among fear of negative evaluation, self-efficacy, mental toughness, and performance anxiety.

**FIGURE 1:**
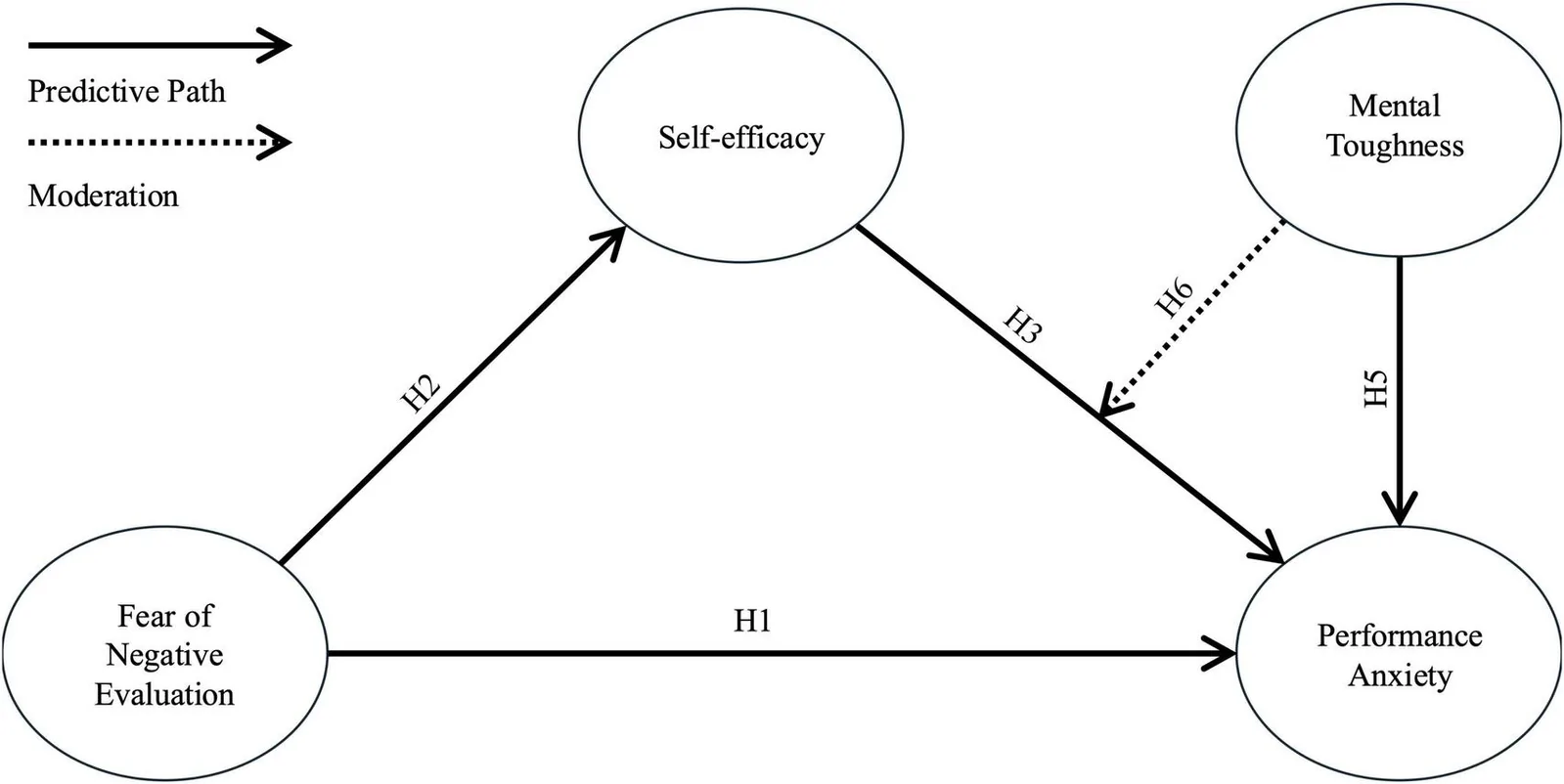
Theoretical framework and hypothesized model. Solid arrows indicate linear main predictive paths; dashed arrows indicate moderating predictive paths. The mediating role of self-efficacy posited in H4 is embedded within the H2 and H3 paths. Asymmetric configurational conditions, such as competitive experience, are not depicted in this linear model diagram.

Complex social-psychological phenomena are the result of the concurrent synergy of multiple antecedent conditions, presenting characteristics of configurational asymmetry and equifinality (Fiss, 2011). This study introduces fuzzy-set qualitative comparative analysis to explore the configurational effects of multiple conditions (Fiss, 2011). Performance anxiety among CUBAL female basketball players is the result of the synergistic interaction of objective experience accumulation, external evaluative pressure, and the absence of internal psychological capital. Accordingly, the following methodological propositions are formulated:

Proposition 1: No single variable (competitive experience, fear of negative evaluation, self-efficacy, or mental toughness) is a necessary condition for the occurrence of high levels of performance anxiety.
Proposition 2: Multiple equifinal sufficient configurational paths explain high performance anxiety, which are formed by the interaction of the presence of external evaluative pressure, the absence of internal psychological resources, and specific experience states.
Proposition 3: The configurational mechanisms linked to performance anxiety exhibit asymmetric characteristics, meaning that the configurational paths associated with high performance anxiety and non-high performance anxiety differ in their core conditional configurations.

## 2 Materials and methods

### 2.1 Measurement instruments

The measurement instruments used in this study were all derived from established scales in the existing literature. To ensure the applicability and semantic equivalence of the foreign scales within the context of CUBAL Division II female basketball players, a standardized translation-back-translation procedure was implemented. Subsequently, cognitive interviews were conducted using a “think-aloud” protocol with a sample from the target population to evaluate and optimize the contextual appropriateness of the items (Willis, 2005). Concurrently, domain experts were invited to assess the content validity of the screened items to establish the final measurement model (Lawshe, 1975). Regarding questionnaire administration, items sharing the same Likert scoring criteria were randomly ordered, effectively controlling for common method bias at the instrument design level.

Performance anxiety was measured using the Sport Anxiety Scale-2 (SAS-2) developed by Smith et al. (2006), retaining its 4-point Likert scale format and all 15 original items. Fear of negative evaluation was assessed using the straightforwardly worded items of the Brief Version of the Fear of Negative Evaluation Scale (BFNE-S), recommended by Rodebaugh et al. (2004) and cross-culturally validated by Pitarch (2010), using all 8 positively worded items on a 5-point Likert scale. Mental toughness was evaluated using the Sports Mental Toughness Questionnaire (SMTQ) developed by Sheard et al. (2009), adhering to the original 4-point scoring criteria and retaining all 14 items.

The measurement of self-efficacy was theoretically grounded in the classic self-efficacy scale developed by Luo (2012), and underwent cross-cultural and situational adaptations based on the Self-Efficacy Scale modified by Zhao et al. (2026) specifically for domestic university basketball league contexts. The measurement of this construct retained 20 items aligned with Division II competitive environments and was evaluated using a 5-point Likert scale.

While this study uses both four-point (i.e., SAS-2 and SMTQ) and five-point (i.e., BFNE-S and Self-Efficacy) Likert response scales, retaining the original response formats of these validated instruments was essential to preserve their established psychometric properties. Construct comparability was ensured, and the use of mixed Likert scales does not threaten the validity of the PLS-SEM estimation, because the PLS-SEM algorithm automatically standardizes all indicator variables (i.e., mean = 0, variance = 1) prior to estimating the structural model. This standardization process effectively eliminates the metric differences among disparate response scales, ensuring unbiased path coefficient estimations (Hair et al., 2019).

### 2.2 Participants and data collection

This study employed a cross-sectional survey design, targeting female basketball players in the Southeast Division of the Chinese University Basketball Association League (CUBAL) Division II as the target population. After obtaining permission from the head coaches of the participating universities and the informed consent of the athletes themselves, data were collected via online questionnaires during April in 2026. To minimize potential common method bias (CMB), several procedural remedies were rigidly adopted during the questionnaire design and administration phases in accordance with Podsakoff et al. (2003). First, to reduce evaluation apprehension, the questionnaire was administered under completely anonymous conditions. Second, the instructions explicitly stated that the data are used for academic research purposes only, and there are no right or wrong answers, which mitigates social desirability effects and encourages honest self-reporting. Finally, to prevent the formation of cognitive response sets, items sharing the same Likert scoring criteria were randomly ordered throughout the survey instrument.

A total of 255 initial questionnaires were collected. To ensure sample homogeneity and data quality, the raw data were screened according to predefined exclusion criteria: first, based on eligibility screening, 84 non-target respondents with backgrounds in athletic admissions, high-level sports teams, or sports-related majors were excluded; second, through response pattern verification, 26 invalid questionnaires displaying straight-lining response patterns (zero variance), highly abnormal completion times, or failure to pass attention checks were removed; finally, through logical screening, 2 invalid responses with contradictory patterns on reverse-coded items (or operational logic conflicts) were excluded. Ultimately, 143 valid samples were obtained, yielding a valid questionnaire recovery rate of 56.1%. To assess the adequacy of this sample size for PLS-SEM, an a priori power analysis was conducted using G*Power 3.1. Assuming a medium effect size (*f*^2^ = 0.15), an alpha level of 0.05, a standard statistical power of 0.80, and a maximum of 7 predictors pointing to the endogenous construct, the minimum required sample size was determined to be 103. The obtained sample of 143 significantly exceeds this methodological threshold, ensuring robust statistical power. Furthermore, regarding the fsQCA, a sample size of 143 falls effectively within the optimal medium-to-large-N range, which provides sufficient case diversity while mitigating the risk of random noise in configurational analysis.

The demographic characteristics of the valid sample are presented in Table 1. The age of the respondents ranged from 18 to 26 years, with a mean age of 20.6 years (SD = 1.73). In terms of grade distribution, the sample spanned from freshmen to postgraduate students in a decreasing distribution. The distribution of court positions and tournament experience was also relatively balanced, indicating that the overall sample possessed adequate representativeness.

**TABLE 1:** Demographic characteristics of the sample (*N* = 143).

Variables	Categories	N	%
Age	18–20	71	49.7
	21–22	49	34.3
	> 23	23	16.1
Grade	Fre.	30	21
	Sop.	41	28.7
	Jun.	33	23.1
	Sen.	24	16.8
	Gra.	15	10.5
Position	Guard	57	39.9
	Forward	55	38.5
	Center	31	21.7
Exp.	1	74	51.7
	2	38	26.6
	3	18	12.6
	≥ 4	13	9.1

*N* = 143. Age includes 18–20, 21–22, > 23. Grade includes Fre, Freshman; Sop, Sophomore; Jun, Junior; Sen, Senior; Gra, Graduate; Position includes Guard, Forward, Center. Exp, Competitive experience (1, 2, 3, or 4 tournament participations).

### 2.3 Pilot study

Subsequently, an item analysis was performed on a small-sample pilot study (*N* = 48), which meets the conventional methodological recommendation of 30–50 participants for initial scale validation. The results indicated that the critical values of all retained items in the item discrimination analysis reached significance levels (*p* < 0.001), and the corrected item-total correlation coefficients were all greater than 0.30, with the sole exception of the “environmental perception dimension” of self-efficacy (see Table 2).

**TABLE 2:** Summary of item analysis and reliability testing for the pilot study.

Construct	Dimension	Item	CR range (t)	CITC	Cronbach’s α
SA	Somatic	5	−8.003 ∼−6.734	0.605 ∼ 0.739	0.843
	Worry	5	−10.976 ∼−7.116	0.672 ∼ 0.810	0.900
	CD	5	−7.097 ∼−5.251	0.630 ∼ 0.762	0.862
SE	Control	4	−6.481 ∼−4.394	0.389 ∼ 0.593	0.675
	Workout	7	−9.622 ∼−6.087	0.674 ∼ 0.775	0.905
	Ability	5	−9.000 ∼−5.117	0.563 ∼ 0.726	0.821
MT	Confidence	6	−12.829 ∼−6.208	0.631 ∼ 0.821	0.893
	Control	5	−7.330 ∼−5.846	0.445 ∼ 0.615	0.737
	Constancy	3	−10.814 ∼−4.514	0.399 ∼ 0.621	0.708
FNE	FNE	8	−14.418 ∼−4.680	0.688 ∼ 0.929	0.955

CR, Critical Ratio; CITC, Corrected Item-Total Correlation; FNE, Fear of negative evaluation; MT, Mental toughness (including Confidence, Control, and Constancy dimensions); SA, Performance anxiety (including Somatic, Worry, and CD dimensions, where CD stands for Concentration disruption); SE, Self-efficacy (including Control, Workout, and Ability dimensions).

Reliability analysis revealed that except for the “environmental perception” dimension of the self-efficacy scale (Cronbach’s α = 0.076), the Cronbach’s α coefficients for all other dimensions ranged between 0.675 and 0.955. Drawing upon Bandura’s self-efficacy theory, high-level athletes’ efficacy beliefs are highly internalized and minimally disrupted by standardized external environments; thus, this dimension failed to demonstrate acceptable internal consistency within the target competitive population and was discarded entirely. The final revised formal measurement instrument comprised 10 dimensions spanning 4 primary constructs (performance anxiety, self-efficacy, mental toughness, and fear of negative evaluation), totaling 53 observed indicators.

### 2.4 Data analysis strategy

This study utilized SPSS 26.0 to conduct descriptive statistics, analysis of variance (ANOVA) for demographic differences, and common method variance (CMB) assessment (Podsakoff et al., 2003).

For the structural model, SmartPLS 4.0 was employed to evaluate the partial least squares structural equation modeling (PLS-SEM). Given that the conceptual model includes higher-order constructs, parameter estimation followed the “Becker’s disjoint two-stage method” (Sarstedt et al., 2019). Upon validating the reliability and validity of the first-order and second-order measurement models, the bootstrapping resampling technique (5,000 resamples) was applied to calculate the statistical significance and effect sizes of the structural path coefficients (Hair et al., 2019).

Additionally, fsQCA 3.0 was utilized to explore the complex configurational mechanisms underlying performance anxiety. Following standard procedural guidelines, continuous raw data were first calibrated into fuzzy-set scores, followed by a necessity analysis for single antecedent conditions (Ragin, 2009). In the sufficiency analysis stage, a truth table was constructed, and by comparing the intermediate and parsimonious solutions, core and peripheral conditions were identified, ultimately elucidating the asymmetric equifinal configurations associated with elevated or mitigated performance anxiety (Fiss, 2011).

A conceptual rationale for using both methods concurrently lies in their complementary analytical capabilities (Pappas and Woodside, 2021). It should be explained that: (a) PLS-SEM is utilized to test the linear relationships, mediating mechanisms, and independent net effects between variables. However, linear models assume symmetric relationships and cannot fully capture the complexity of human psychological phenomena. Therefore, (b) a configurational approach (fsQCA) is necessary to identify the complex combinations of conditions (configurations) that lead to specific outcomes. Ultimately, (c) the two methods complement each other, thereby providing a more comprehensive understanding than using a single approach alone. PLS-SEM establishes the baseline predictive validity, while fsQCA reveals the asymmetric, equifinal pathways.

## 3 Results

### 3.1 Common method bias testing

The core data in this study relied on self-report questionnaires. To avoid the potential impact of common source variance, Harman’s single-factor test was employed to assess common method bias (CMB). All 53 observed indicators involving all latent variables were entered into an unrotated exploratory factor analysis. The results indicated that the variance explained by the first principal factor was 24.830%, which is well below the conventional threshold of 40%. Thus, it was determined that the empirical data in this study did not suffer from severe common method bias, meeting the data quality requirements for subsequent multivariate statistical and configurational analyses.

To further enhance the rigor of the CMB testing, this study introduced the full collinearity assessment approach for post hoc verification, following the methodological recommendations of Kock (2015) (detailed full collinearity VIF results are provided in section 4.3.2). Since the variance inflation factors of all core variables in the model were below the conservative critical threshold of 3.3, the interference of common method bias was ruled out at the structural model level (Kock, 2015).

### 3.2 Descriptive statistics and demographic differences

The descriptive statistics and correlation analysis results of the core variables in this study are presented in Table 3. The surveyed athlete population overall exhibited data characteristics of “relatively high self-efficacy (*M* = 3.904), moderately high fear of negative evaluation (*M* = 3.139), and moderately low performance anxiety (*M* = 1.955).” Pearson correlation tests indicated significant correlations (*p* < 0.01) among all core constructs, with directions aligning with theoretical expectations.

**TABLE 3:** Descriptive statistics and correlation matrix of the core variables (*N* = 143).

Constructs	Mean	SD	1. FNE	2. SE	3. MT	4. SA
1 FNE	3.139	0.905	1	1	1	1
2 SE	3.904	0.471	−0.226**			
3 MT	2.765	0.375	−0.562***	0.440***		
4 SA	1.955	0.56	0.518***	−0.468***	−0.629***	

FNE, Fear of negative evaluation; MT, Mental toughness; SA, Performance anxiety; SE, Self-efficacy. Values below the diagonal are Pearson correlation coefficients among latent variables.

**p < 0.01, ***p < 0.001.

The results of the one-way analysis of variance (ANOVA) are shown in Table 4. Age and court position had no significant main effects on any of the core variables. Grade level only showed a significant difference in self-efficacy (*F* = 2.571, *p* < 0.05), and LSD post hoc comparisons revealed that the self-efficacy of seniors and graduate students was significantly higher than that of sophomores and juniors.

**TABLE 4:** Differences in core variables across demographic characteristics (*N* = 143).

Constructs	Group	N	FNE	SE	MT	SA
Age			1.052	1.617	0.856	0.000
	18-20	71	3.21 ± 0.93	3.86 ± 0.48	2.73 ± 0.37	1.95 ± 0.50
	21-22	49	2.99 ± 0.94	3.90 ± 0.45	2.79 ± 0.41	1.96 ± 0.64
	≥ 23	23	3.23 ± 0.74	4.06 ± 0.47	2.83 ± 0.31	1.96 ± 0.58
Grade			1.410	2.571*	1.203	0.952
	Fre.	30	3.30 ± 0.99	3.98 ± 0.48	2.75 ± 0.29	1.94 ± 0.38
	Sop.	41	3.05 ± 0.91	3.78 ± 0.42	2.71 ± 0.37	1.94 ± 0.62
	Jun.	33	3.27 ± 0.87	3.81 ± 0.45	2.72 ± 0.46	2.11 ± 0.55
	Sen.	24	2.82 ± 0.83	4.04 ± 0.52^b^	2.90 ± 0.37	1.86 ± 0.64
	Gra.	15	3.32 ± 0.85	4.10 ± 0.47^b,c^	2.81 ± 0.35	1.85 ± 0.57
Position			0.239	0.155	0.524	0.793
	Guard	57	3.07 ± 0.89	3.92 ± 0.46	2.79 ± 0.38	1.90 ± 0.51
	Forward	55	3.18 ± 0.88	3.88 ± 0.45	2.77 ± 0.33	1.96 ± 0.54
	Center	31	3.19 ± 0.99	3.93 ± 0.54	2.71 ± 0.44	2.06 ± 0.67
EXP			4.061**	1.923	4.073**	4.450**
	1	74	3.27 ± 0.97	3.86 ± 0.45	2.71 ± 0.34	2.00 ± 0.49
	2	38	3.25 ± 0.73	3.84 ± 0.48	2.73 ± 0.36	2.09 ± 0.65
	3	18	2.87 ± 0.72	4.02 ± 0.53	2.82 ± 0.45	1.84 ± 0.58
	≥ 4	13	2.44 ± 0.93^ab^	4.15 ± 0.43	3.08 ± 0.36^a,b,c^	1.49 ± 0.42^a,b^

Age includes 18-20, 21–22, > 23. Grade includes Fre.-Freshman, Sop, Sophomore; Jun, Junior; Sen, Senior; Gra, Graduate. Position includes Guard, Forward, Center. Exp, Competitive experience (1, 2, 3, or 4 tournament participations).

*p < 0.05, **p < 0.01. Groups sharing the same superscript letter indicate significant differences in LSD post hoc comparisons (*p* < 0.05), where “a” denotes a significant difference from Group 1, “b” denotes a significant difference from Group 2, and “c” denotes a significant difference from Group 3.

The number of tournament participations exhibited significant between-group differences in fear of negative evaluation, mental toughness, and performance anxiety. Post hoc comparisons indicated that athletes with four tournament participations had significantly lower levels of fear of negative evaluation and performance anxiety compared to the novice group with one or two participations, while scoring the highest in mental toughness. Based on the results of the above difference analysis, the subsequent structural equation model incorporated “number of tournament participations” and “grade” transformed into dummy variables as control variables to account for potential confounding effects (Bernerth and Aguinis, 2016).

### 3.3 Measurement model evaluation

This study employed partial least squares structural equation modeling (PLS-SEM) to evaluate the measurement model. Given that the model included higher-order constructs, this study adopted the “Becker’s disjoint two-stage method” to extract the first-order latent variable scores to construct the second-order measurement model (Sarstedt et al., 2019). The reliability and convergent validity test results for the first-order and second-order measurement models are detailed in Table 5.

**TABLE 5:** Reliability and convergent validity of the measurement model.

Constructs and dimensions	1st-order loadings	2nd-order loadings	Cronbach’s α	CR (rho_c)	AVE
FNE	0.772–0.881	–	0.931	0.943	0.675
MT	–	–	0.57	0.774	0.541
Confidence	0.63–0.786	0.571	0.825	0.873	0.535
Constancy	0.58–0.939	0.708	0.42	0.747	0.61
Control	0.627–0.817	0.892	0.752	0.842	0.574
SA	–	–	0.833	0.898	0.747
Somatic	0.73–0.878	0.864	0.866	0.902	0.65
Worry	0.845–0.882	0.869	0.915	0.936	0.746
CD	0.712–0.808	0.86	0.814	0.871	0.574
**SE**	–	–	0.79	0.863	0.678
Control	0.709–0.816	0.77	0.665	0.815	0.595
Workout	0.620–0.873	0.828	0.815	0.864	0.518
Ability	0.686–0.769	0.869	0.562	0.766	0.523

FNE, Fear of negative evaluation; MT, Mental toughness (including Confidence, Control, and Constancy dimensions); SA, Performance anxiety [including Somatic, Worry, and CD (Concentration disruption) dimensions]; SE–Self-efficacy (including Control, Workout, and Ability dimensions); CR, Composite Reliability; AVE, Average Variance Extracted. Given the higher-order construct design of this study, first- and second-order measurement parameters are presented cumulatively in the table. A few dimensions with slightly lower Cronbach’s α values were retained to preserve content validity, as their CR and AVE values met the acceptable standards.

#### 3.3.1 Reliability and convergent validity assessment

In the initial measurement model assessment stage, this study screened the observed variables based on the indicator purification procedure by Hair et al. First, low-loading indicators with factor loadings below 0.40 were deleted. Second, for indicators with loadings between 0.40 and 0.70, six items that significantly undermined the convergent validity of their respective constructs were deleted, provided that the content validity of the scale was not compromised. Following this purification procedure, the average variance extracted (AVE) of all constructs reached the theoretical threshold of 0.50 (Hair et al., 2019).

Regarding indicator reliability, the standardized factor loadings of most first-order and second-order indicators were above the recommended threshold of 0.70. The factor loadings of some first-order indicators ranged between 0.581 and 0.700; however, since deleting these indicators failed to increase the AVE of their respective constructs above the critical value, and to maintain the content validity of the constructs, they were retained (Hair et al., 2019).

In terms of internal consistency reliability, this study reported both Cronbach’s α and composite reliability (CR). Although the Cronbach’s α for certain first-order dimensions (e.g., the Constancy dimension of mental toughness) was below 0.70, given that Cronbach’s α assumes equal weighting of indicators and is sensitive to the number of items, CR provides a more robust reliability assessment in PLS-SEM (Hair et al., 2019). The results showed that the CR values for all first-order and second-order constructs ranged from 0.747 to 0.943, meeting the acceptable criterion of being greater than 0.70, confirming that the model possessed good internal consistency.

Regarding convergent validity, the AVE values of all first-order dimensions and second-order main constructs ranged from 0.518 to 0.747, which are higher than the criterion threshold of 0.50, indicating that each latent variable could explain more than half of the variance of its indicators, demonstrating good convergent validity of the model.

#### 3.3.2 Discriminant validity testing

The discriminant validity of the first-order dimension measurement model was assessed using the Heterotrait-Monotrait ratio of correlations (HTMT), and the results are shown in Table 6. Except for a few combinations, the HTMT values between most constructs were below the 0.85 standard (Henseler et al., 2015). The HTMT value between the lower-order dimensions of self-efficacy, SE-Control and SE-workout, was 1.068, exceeding the traditional threshold. The high covariation (HTMT > 1.0) presented in the empirical data indicates that they are highly homologous in the target group’s cognition, essentially measuring the homogeneous core of the same latent variable. According to the norms for higher-order constructs (HOCs) by Sarstedt et al. (2019), a high degree of overlap among lower-order dimensions under the same reflective higher-order construct not only aligns with theoretical settings but is also a necessary statistical feature for establishing the convergent validity of the higher-order construct; thus, it is not considered a violation of discriminant validity. Overall, the quality of the measurement model met the standards, supporting the subsequent structural model testing.

**TABLE 6:** Heterotrait-Monotrait ratio (HTMT) matrix for first-order dimensions.

Constructs/ Dimensions	1	2	3	4	5	6	7	8	9	10
1. FNE	–	–	–	–	–	–	–	–	–	–
2. MT-Confidence	0.211									
3. MT-Constancy	0.378	0.34								
4. MT-Control	0.761	0.371	0.831							
5. SA-Somatic	0.321	0.289	0.375	0.514						
6. SA-Worry	0.682	0.322	0.42	0.822	0.675					
7. SA- CD	0.389	0.383	0.61	0.546	0.822	0.652				
8. SE-Control	0.14	0.787	0.221	0.233	0.334	0.243	0.434			
9. SE-Workout	0.111	0.737	0.192	0.158	0.286	0.166	0.417	1.068		
10. SE-Ability	0.393	0.614	0.461	0.536	0.566	0.568	0.607	0.718	0.814	

FNE, Fear of negative evaluation; MT, Mental toughness (Confidence, Control, Constancy); SA, Performance anxiety [Somatic, Worry, CD(Concentration disruption)]; SE, Self-efficacy (Control, Workout, Ability); HTMT, Heterotrait-Monotrait ratio of correlations. According to conventional standards, HTMT values below 0.85 (or the conservative threshold of 0.90) across principal construct dimensions indicate good discriminant validity. Certain combinations exceeding the threshold in the table (e.g., Workout and Control) are subordinate to the same higher-order latent variable (Self-efficacy), presenting a high degree of conceptual overlap; their high HTMT values align with the theoretical expectations and statistical logic of higher-order model specifications.

The discriminant validity of the second-order measurement model was tested using the HTMT ratio, as shown in Table 7. The highest HTMT value among all second-order constructs was 0.856, which is below the 0.90 critical value, confirming good discriminant validity among the higher-order latent variables (Henseler et al., 2015).

**TABLE 7:** Heterotrait-Monotrait ratio (HTMT) matrix for second-order constructs.

Constructs	1. FNE	2. MT	3. SA	4. SE
1 FNE	–	–	–	–
2 MT	0.665			
3 SA	0.54	0.856		
4 SE	0.193	0.677	0.53	

FNE, Fear of negative evaluation; MT, Mental toughness; SA, Performance anxiety; SE, Self-efficacy; HTMT, Heterotrait-Monotrait ratio of correlations. According to conventional standards, HTMT values below 0.85 (or the conservative threshold of 0.90) across principal construct dimensions indicate good discriminant validity. Certain combinations exceeding the threshold in the table (e.g., Workout and Control) are subordinate to the same higher-order latent variable (Self-efficacy), presenting a high degree of conceptual overlap; their high HTMT values align with the theoretical expectations and statistical logic of higher-order model specifications.

The collinearity test results revealed that the variance inflation factors (VIF) of all predictor variables ranged from 1.000 to 1.740, well below the threshold of 3.0, indicating the absence of severe collinearity issues in the model (Hair et al., 2019).

### 3.4 Structural model path hypothesis testing

The assessment of the model’s explanatory power showed that the explained variance rates for performance anxiety (SA) and self-efficacy (SE) were 50.0 and 5.1%, respectively. The significance and 95% bias-corrected confidence intervals for the direct, indirect, and moderating effects were tested using a bootstrapping procedure with 5,000 resamples, with detailed results in Table 8.

**TABLE 8:** Structural equation model path coefficients and hypothesis testing.

Path	β	*T*	95% CI	*f* ^2^
Direct effect				
FNE →SA	0.261***	3.923	[0.135, 0.397]	0.093
FNE →SE	−0.226**	2.893	[−0.357, −0.046]	0.054
SE →SA	−0.234***	3.675	[−0.351, −0.101]	0.088
MT →SA	−0.400***	5.096	[−0.548, −0.239]	0.184
MT × SE →SA	0.100*	1.999	[−0.002, 0.191]	0.034
Specific Indirect effect				
FNE →SE →SA	0.053*	2.286	[0.015, 0.102]	–

FNE, Fear of negative evaluation; MT, Mental toughness; SA, Performance anxiety; SE, Self-efficacy. β is the standardized path coefficient; CI is the 95% bias-corrected confidence interval (calculated based on 5,000 bootstrapping resamples).

*p < 0.05, **p < 0.01, ***p < 0.001.

Main effect testing: Fear of negative evaluation significantly and positively predicted performance anxiety and significantly and negatively predicted self-efficacy. Self-efficacy significantly and negatively predicted performance anxiety.

Moderating effect testing: Mental toughness demonstrated a significant negative predictive relationship with performance anxiety. The path coefficient for the interaction term (MT × SE) on performance anxiety was 0.100 (*p* = 0.046), indicating that mental toughness played a significant moderating role between self-efficacy and performance anxiety.

Mediating effect testing: The specific indirect effect of fear of negative evaluation on performance anxiety through self-efficacy was significant, and the 95% confidence interval ([0.015, 0.102]) did not include 0, confirming the partial mediating role of self-efficacy.

To rule out the potential interference of sample demographic differences on core path estimations, this study, based on the ANOVA results, transformed “grade” and “number of tournament participations”—which showed significant between-group differences—into dummy variables and included them as control variables in the structural model. Specifically, “freshman” was used as the reference group for grade, and “1 participation” was the reference group for the number of tournament participations.

The estimation results of the structural model including control variables are detailed in Table 9 and visually summarized in Figure 2. After incorporating the control variables, the core main effects and moderating effects of the model remained robust: the positive effect of fear of negative evaluation on performance anxiety and its negative effect on self-efficacy remained significant; the negative predictive role of self-efficacy on performance anxiety and the moderating role of mental toughness were also significant. The model’s explained variance rates for performance anxiety (SA) and self-efficacy (SE) were 50.9 and 11.2%, respectively. This confirms that the core theoretical framework of this study possesses high cross-sample robustness and is not substantially confounded by the accumulation of experience or grade progression.

**TABLE 9:** Structural model path analysis results including control variables.

Path	β	*T*	95%CI	*f* ^2^
Direct effects				
FNE→SA	0.246***	3.606	[0.116, 0.379]	0.081
FNE→ SE	−0.184*	2.167	[−0.346, −0.014]	0.033
SE →SA	−0.216**	3.129	[−0.348, −0.073]	0.075
MT→ SA	−0.395***	4.850	[−0.553, – 0.234]	0.181
MT × SE → SA	0.111*	2.196	[0.008, 0.204]	0.041
Control variables effects				
Sop. → SE	−0.499*	2.068	[−0.962, −0.021]	0.031
Jun. → SE	−0.489	1.775	[−1.035, 0.050]	0.022
Sen. → SE	−0.232	0.538	[−1.056, 0.621]	0.003
Gra. → SE	−0.037*	0.082	[−0.984, 0.789]	0
2 Times → SA	0.187	1.1	[−0.133, 0.523]	0.013
3 Times → SA	−0.041	0.22	[−0.386, 0.361]	0
4 times → SA	−0.242	1.38	[−0.582, 0.100]	0.008
2 Times → SE	0.127	0.511	[−0.376, 0.586]	0.002
3 Times → SE	0.259	0.705	[−0.451,0.975]	0.005
4 Times → SE	0.434	0.931	[−0.476,1.334]	0.009
Specific indirect effect				
FNE → SE → SA	0.04	1.749	[0.006,0.097]	–

FNE, Fear of negative evaluation; MT, Mental toughness; SA, Performance anxiety; SE, Self-efficacy. Among the control variables, “Freshman” serves as the reference group for Grade, and “1 participation” serves as the reference group for Competitive experience.

*p < 0.05, **p < 0.01, ***p < 0.001. The significance of indirect effects is determined by whether the 95% bootstrapping bias-corrected confidence interval includes 0.

**FIGURE 2:**
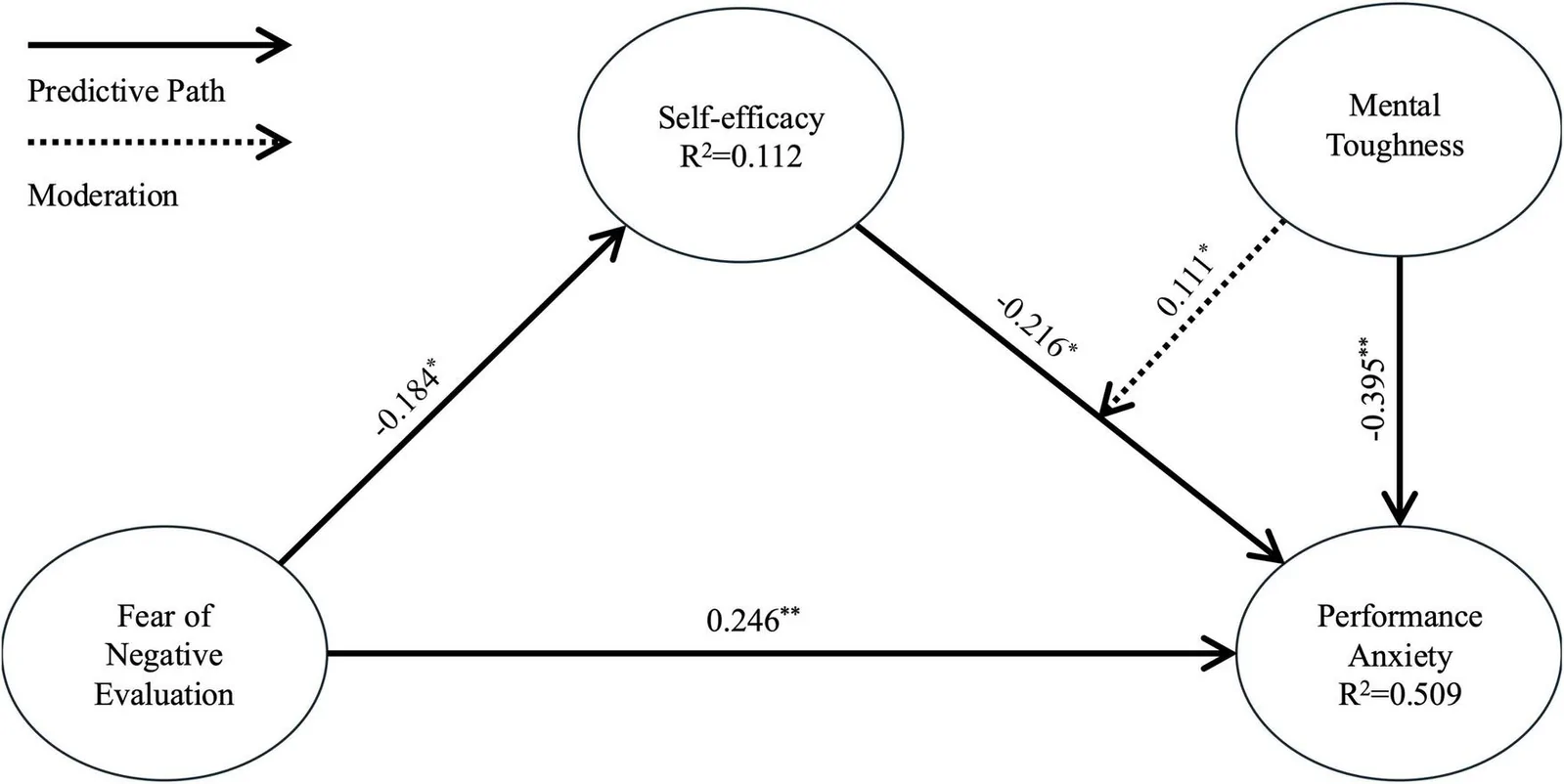
Structural model testing results. Standardized path coefficients (β) are reported. *p < 0.05, **p < 0.01. FNE, Fear of negative evaluation; SE, Self-efficacy; MT, Mental toughness; SA, Performance anxiety. The path coefficients and explained variance (R^2^) represent the estimations from the full structural model, including control variables (grade and competitive experience). For visual clarity, the control variables and their respective paths are omitted from this diagram. Please refer to Table 9 for the complete statistical results.

Among the control variable paths, only the sophomore grade showed a significant negative association with self-efficacy (β = -0.499, *p* < 0.05), while the remaining control paths did not reach significance levels. Furthermore, although the control variables explained a portion of the covariance, and the *t*-test based on traditional parameter assumptions decreased slightly, the specific indirect effect based on 5,000 bootstrapping resamples (FNE - > SE - > SA, β = 0.040) yielded a 95% bias-corrected confidence interval of [0.006, 0.097]. According to the criteria for nonparametric testing in PLS-SEM, since the 95% confidence interval did not include 0, the partial mediating role of self-efficacy remained significant after controlling for demographic variables (Hair et al., 2019).

This further indicates that exploring net effects between variables in isolation within a linear model has limitations, providing a methodological rationale for subsequently employing fsQCA to explore the nonlinear configurational paths of multiple variables synergistically associated with performance anxiety.

### 3.5 Fuzzy-set qualitative comparative analysis (fsQCA)

To explore the nonlinear and synergistic effects of competitive experience, fear of negative evaluation, mental toughness, and self-efficacy on athletes’ performance anxiety, this study employed the fsQCA method for configurational analysis (Ragin, 2009).

#### 3.5.1 Data calibration

Prior to the configurational analysis, this study calibrated the raw data of the antecedent conditions and the outcome variable into fuzzy-set membership scores ranging from 0 to 1 (Ragin, 2009). Addressing the differences in the measurement properties of the variables, two calibration strategies were implemented.

For continuous scale variables such as performance anxiety, fear of negative evaluation, mental toughness, and self-efficacy, this study utilized the percentile method to define anchor points based on objective data distribution: the 95th, 50th (median), and 5th percentiles of the sample data were set as the thresholds for full membership, the cross-over point, and full non-membership, respectively (Fiss, 2011). To prevent cases from being systematically dropped during the truth table algorithm due to calibration scores exactly hitting 0.5, a fine-tuning adjustment of 0.001 was added to all values of 0.5 in the calibration procedure (Fiss, 2011).

For the objective ordinal variable “competitive experience,” based on its progressive four-point scale characteristics (1 participation to 4 or more participations), the direct assignment method was used to map its original categories into specific fuzzy-set membership scores: these were set to 0.05 (full non-membership), 0.33 (mostly but not fully non-membership), 0.67 (mostly but not fully membership), and 0.95 (full membership) respectively, to accurately reflect the differences in the degree of membership across experience levels (Ragin, 2009).

#### 3.5.2 Necessity analysis

Before constructing the configurations, it was first tested whether any single antecedent variable was a necessary condition for the outcome variable. The highest necessity consistency for all single antecedent conditions was 0.727, none of which reached the judgment threshold of 0.90 (Schneider and Wagemann, 2012). This indicates that no single variable alone constitutes a necessary condition for high performance anxiety; rather, the emergence of performance anxiety is a configurational outcome of the concurrent effects of multiple factors.

#### 3.5.3 Sufficiency configurational analysis and configurational asymmetry testing

To effectively circumvent simultaneous subset relations and ensure the reliability of configurational inferences, this study set the Proportional Reduction in Inconsistency (PRI) threshold to a stringent 0.70, aligning with expert methodological recommendations (Greckhamer et al., 2018; Pappas and Woodside, 2021). After eliminating logical remainders with PRI below 0.70, the raw consistency thresholds for high performance anxiety (SA) and non-high performance anxiety (∼SA) were established at 0.893 and 0.874 respectively, based on natural breaks in the data distribution, both of which are higher than the conventional benchmark of 0.80 (Fiss, 2011). The case frequency threshold was set at 1.

During the counterfactual analysis stage for constructing the intermediate solution, directional expectations were set based on theoretical deductions: for the positive set prediction of high performance anxiety, fear of negative evaluation was set as “present,” while mental toughness and self-efficacy were set as “absent”; for the negative set prediction of non-high performance anxiety, fear of negative evaluation was set as “absent,” while mental toughness and self-efficacy were set as “present.” Due to the controversy regarding the dual effects of competitive experience, it was set as either “present or absent” in both positive and negative set predictions. The computational results showed that the intermediate solutions extracted from the high and low anxiety models were identical to the parsimonious solutions. According to the criteria established by Fiss (2011), there were no peripheral conditions in the models; all identified antecedent variables were entirely defined as core conditions. The merged results of the configurational analysis are presented in Table 10.

**TABLE 10:** Sufficiency configurational analysis results for performance anxiety.

Conditions	SA		∼SA
Antecedent conditions	H1	H2	L1
EXP	●		
FNE		●	⊗
MT	⊗	⊗	●
**SE**			●
Model fit metrics			
Consistency	0.92	0.909	0.884
Raw coverage	0.375	0.651	0.582
Unique coverage	0.021	0.297	0.582
Solution consistency		0.901	0.884
Solution coverage		0.672	0.582

FNE, Fear of negative evaluation; MT, Mental toughness; SA, Performance anxiety; SE, Self-efficacy; EXP, Competitive experience. ● indicates the presence of a core condition; ⊗ indicates the absence of a core condition; blank spaces indicate that the presence or absence of the condition in the configuration does not significantly affect the outcome. Given that the intermediate and parsimonious solutions extracted for both the high and low anxiety sets in this model are identical, all conditions listed in the table are defined as core conditions. The PRI consistency thresholds during the calculations for both high anxiety (SA) and non-high anxiety (∼SA) were set to 0.70.

As shown in Table 10, there are 2 equifinal configurations leading to high performance anxiety (H1, H2), with an overall solution consistency of 0.901 and an overall solution coverage of 0.672.

Configuration H1 (EXP * ∼MT) indicates that the concurrence of high competitive experience and the absence of mental toughness constitutes a sufficient condition for high performance anxiety. Configuration H2 (FNE * ∼MT) implies that high fear of negative evaluation concurrent with the absence of mental toughness constitutes a sufficient condition for high performance anxiety. Comparing the two paths, the scarcity of mental toughness (∼MT) is a common core condition linked to high performance anxiety. When individuals lack this psychological resource, either high competitive experience (H1) or high fear of negative evaluation (H2) can serve as a sufficient condition for elevated performance anxiety.

There is 1 configuration associated with non-high performance anxiety (L1), with an overall solution consistency of 0.884 and an overall solution coverage of 0.582. Configuration L1 (∼FNE * MT * SE) indicates that the concurrent absence of fear of negative evaluation, high mental toughness, and high self-efficacy constitutes a sufficient condition for averting performance anxiety. Comparing the configurational features of high and non-high performance anxiety reveals that averting performance anxiety (L1) requires the absence of external environmental pressure concurrent with the presence of internal psychological resources; conversely, the configurations for high performance anxiety (H1, H2) only require the absence of a single psychological resource. The high and non-high anxiety paths lack a symmetric mapping relationship in their condition compositions, confirming the asymmetric characteristic of the configurational mechanisms linked to performance anxiety (Fiss, 2011; Woodside, 2014).

#### 3.5.4 Robustness analysis

To verify the reliability of the configurational analysis results, this study executed robustness checks by adjusting the data calibration anchor points (Fiss, 2011; Greckhamer et al., 2018). The full non-membership and full membership anchor points for continuous variables were contracted inward from the 5th and 95th percentiles to the 10th and 90th percentiles; the fuzzy-set membership assignments for the ordinal variable “competitive experience” were fine-tuned from (0.05, 0.33, 0.67, 0.95) to (0.10, 0.40, 0.60, 0.90). This parameter adjustment compressed the extreme value distribution of the fuzzy sets while adhering to the set-theoretic principle of not crossing the qualitative cross-over point (0.5).

The calculations were re-executed while maintaining the PRI consistency threshold (0.70) and the case frequency threshold (1) unchanged. The results showed that the configurations for high performance anxiety generated after adjusting the anchor points (FNE * ∼MT * ∼SE and EXP * FNE * ∼MT) were both true subsets of the original core configurations (EXP * ∼MT and FNE * ∼MT). Among the newly generated equifinal configurations for non-high performance anxiety, one (∼FNE * MT * SE) was identical to the original path, and the other (∼EXP * ∼FNE * SE)—the concurrence of absent competitive experience, absent fear of negative evaluation, and high self-efficacy—was highly isomorphic in its condition composition with the original conclusion. The overall solution consistencies before and after the adjustment (0.923 for high performance anxiety and 0.852 for non-high performance anxiety) both met the theoretical judgment criteria, and the directions and combinatory logic of the core conditions did not undergo substantive changes. According to the criteria for configurational robustness, clear subset relationships and substantive isomorphism exist between the new and original solutions, confirming that the configurational inferences of this study were not disrupted by the contingency of specific calibration parameters, demonstrating that the model possesses a high degree of robustness (Schneider and Wagemann, 2012).

## 4 Discussion

### 4.1 Discussion of results

The current study presents novel empirical evidence regarding the mechanisms of competitive anxiety within the specific resource-constrained context of CUBAL Division II athletes. Moving beyond the mere replication of existing linear models, our structural equation modeling (SEM) results elucidate a dual-mechanism of internal psychological capital. Specifically, we found that external evaluative pressure does not solely trigger anxiety; rather, it severely disrupts athletes’ self-competence appraisal, manifesting as a sharp decline in self-efficacy, which in turn inevitably translates into elevated performance anxiety (Chen et al., 2026). In this “academic-training” dual-role conflict environment where professional psychological support is structurally lacking, our data robustly demonstrate that mental toughness functions not merely as a trait, but as a critical meta-resource (β = 0.111, *p* < 0.05). It provides a compensatory buffering mechanism that halts the downward spiral of resource depletion, allowing female athletes to maintain emotional stability despite severe fluctuations in self-efficacy. This context-specific finding expands upon classical theories by confirming how positive psychological resources operate defensively under objective constraints (Hobfoll, 1989; Kristjánsdóttir et al., 2018).

Furthermore, the fuzzy-set qualitative comparative analysis (fsQCA) fundamentally shatters the conventional linear assumption that competitive experience is universally protective. The sufficiency configurational analysis identified the high performance anxiety path H1 (EXP * ∼MT), explicitly indicating that the concurrence of high competitive experience and the absence of mental toughness constitutes a sufficient condition for high performance anxiety. This specific configurational finding derived directly from our data provides a new perspective on competitive stress. It suggests that as experience accumulates, the accompanying role expectations and identity maintenance pressures transform into severe cognitive loads for senior athletes. Only when these internal resources are profoundly depleted (∼MT) does objective experience morph into a maladaptive risk factor (Arnold and Fletcher, 2012). This empirical revelation from our CUBAL sample strongly resonates with the recent theoretical propositions regarding the synergistic interactions of pressure and psychological traits (Dziuba et al., 2025), ultimately proving that experience is a double-edged condition rather than an absolute shield. In contrast, the non-high performance anxiety configuration L1 (∼FNE * MT * SE) demonstrates that averting performance anxiety requires the absence of external evaluative pressure concurrent with the presence of dual internal psychological capital. This differs from the core sufficient pathways of high performance anxiety configurations, which only require the absence of a single psychological resource (∼MT), thereby confirming the configurational asymmetry characteristics of the performance anxiety mechanism (Fiss, 2011; Woodside, 2014).

Integrating partial least squares structural equation modeling with fuzzy-set qualitative comparative analysis constructed a comprehensive analytical framework that combines symmetric and asymmetric approaches (Woodside, 2014; Pappas and Woodside, 2021). The SEM established the baseline linear relationships among fear of negative evaluation, psychological capital, and performance anxiety; however, when handling the complex competitive context where multiple factors intertwine, the linear model exhibited localized limitations in explanatory power due to the variance-absorbing effects of control variables (Bernerth and Aguinis, 2016). Conversely, fsQCA, through nonlinear combinations, elucidated the heterogeneous configurational roles of competitive experience and external pressure under different psychological capital configurations. This dual-analytical design addresses the inherent limitations of traditional statistical models that assume variables are independent and compete to explain variance. Within the specific context of female student-athletes facing resource constraints, it more comprehensively delineates the synergistic effects between external social evaluation and internal psychological capital, thereby enhancing the theoretical explanatory power of the antecedent mechanisms of performance anxiety.

### 4.2 Theoretical implications

From a theoretical perspective, this study expands the methodological boundaries and complexity frameworks of competitive emotion research. Traditional sports psychology frameworks predominantly rely on linear symmetric models to evaluate the independent net effects of antecedent variables on competitive anxiety (Hair et al., 2019). By introducing complexity theory and the configurational analysis paradigm, this study confirms that the occurrence and mitigation of performance anxiety possess characteristics of configurational asymmetry (Fiss, 2011; Woodside, 2014). The research demonstrates that no single psychological trait or external characteristic constitutes a necessary condition for the occurrence of anxiety; rather, performance anxiety is a configurational outcome of the dynamic synergy of multiple variables. This provides a theoretical supplement from a nonlinear perspective for understanding the etiology of emotion in complex competitive stress contexts (Mellalieu et al., 2009; Pappas and Woodside, 2021).

Regarding the delineation of variable boundaries, this study advances the understanding of the boundary conditions of “competitive experience” in the sports arena. Existing literature generally views competitive experience as a protective linear variable that suppresses competitive anxiety (Martens et al., 1990). The configurational analysis in this study demonstrates that when high competitive experience intertwines with insufficient mental toughness or high fear of negative evaluation, it constitutes a sufficient condition for high performance anxiety. This finding supplements cognitive appraisal theory, indicating that the role expectations and potential pressure accompanying experience accumulation often transform into cognitive load, suggesting that future emotion prediction models should treat competitive experience as a conditional variable with heterogeneous configurational effects rather than a universally protective factor (Hanin, 2007; Arnold and Fletcher, 2012).

Furthermore, this study uncovers the synergistic and compensatory mechanisms of positive psychological capital within environments characterized by specific resource constraints. The research not only verified the moderating effect of mental toughness on self-efficacy at the linear level but also established the strong compensatory path of self-efficacy and mental toughness for experiential disadvantages at the configurational level (Luthans et al., 2015). The results show that for novice athletes lacking practical competitive experience, the concurrent presence of high mental toughness and high self-efficacy constitutes a sufficient condition for averting performance anxiety. This enriches the applicability of positive psychological capital theory in the sports domain and establishes the core moderating role of psychological capital in buffering the internalization of external evaluative pressure into negative emotions (Bandura, 1997; Gucciardi et al., 2015).

### 4.3 Practical implications

Coaches and management teams are advised to reform the evaluation mechanisms of sports teams to control athletes’ fear of negative evaluation. In daily training and competition assessments, a task-mastery-oriented motivational climate should be cultivated, diminishing performance-based evaluations that rely solely on match outcomes. By guiding athletes to focus their attention on tactical execution and the self-improvement of technical details, the negative impact of external evaluative pressure on performance anxiety can be mitigated (Smith et al., 2007). Specifically, coaches should implement process-oriented feedback protocols (e.g., implementing post-game video reviews focused strictly on tactical adherence and effort markers rather than scoreboard outcomes) to decouple athletes’ self-worth from absolute match results.

The management team should consider differentiated psychological load monitoring for senior athletes with high competitive experience. Given that high competitive experience can transform into an anxiety risk condition under specific configurations, the coaching staff needs to monitor the psychological load brought about by role expectations and identity maintenance among core team members (Arnold and Fletcher, 2012). Actionable steps must be taken to alleviate this specific configuration of anxiety. Before major tournaments, team managers should conduct cognitive reframing workshops specifically for senior players. These sessions should explicitly focus on expectation management and decoupling their leadership identity from the necessity of flawless execution. By formally granting senior athletes the “permission to make mistakes” without losing their core status, coaches can prevent the maladaptive transformation of experience into cognitive load.

The cultivation of positive psychological capital needs to be integrated early into the developmental pathways for young athletes. Because high self-efficacy and high mental toughness can effectively offset a lack of practical experience, training programs should incorporate psychological skills training and contemporary psychological interventions specifically tailored for lower-grade or first-time novice athletes (Ptáček et al., 2026). Actionable recommendations for grassroots teams include pairing novices with highly resilient senior players in a structured peer-mentoring system (vicarious reinforcement), and deliberately introducing ‘noise and referee error’ during practice scrimmages (stress scenario simulation). These specific interventions accelerate the accumulation of psychological capital, equipping young athletes with the necessary buffer to offset their lack of practical competitive experience.

## 5 Conclusion

Based on self-presentation theory and the positive psychological capital framework, this study adopted a dual-analytical approach combining partial least squares structural equation modeling (PLS-SEM) and fuzzy-set qualitative comparative analysis (fsQCA) to explore the synergistic influence mechanisms of fear of negative evaluation, self-efficacy, mental toughness, and competitive experience on the performance anxiety of 143 CUBAL Division II female basketball players. The SEM analysis indicated that fear of negative evaluation positively predicted performance anxiety and negatively predicted self-efficacy, while mental toughness negatively predicted performance anxiety and significantly moderated the relationship between self-efficacy and performance anxiety. The fsQCA further revealed characteristics of configurational asymmetry, wherein the concurrence of high competitive experience and the absence of mental toughness constituted a sufficient condition for explaining high performance anxiety, whereas the concurrence of absent fear of negative evaluation, high mental toughness, and high self-efficacy formed a sufficient path for averting performance anxiety. This study clarifies the nonlinear boundary of how competitive experience affects performance anxiety, elucidates the nonlinear configurational mechanisms of the synergistic effects of multidimensional internal and external factors, and provides empirical evidence for collegiate sports programs to implement targeted psychological interventions.

Ultimately, the originality of this study lies in its methodological and theoretical shift from traditional linear paradigms to a configurational perspective, providing the first empirical evidence of how external evaluative pressure and internal psychological capital asymmetrically synergize to precipitate or avert anxiety in a resource-constrained athletic cohort. Furthermore, this study significantly contributes to future research by establishing a dual-analytical blueprint. It demonstrates that subsequent emotion prediction models in sports psychology must conceptualize competitive experience and psychological traits not as isolated protective predictors, but as fundamentally interdependent, configurational variables.

### 5.1 Limitations and future research directions

Although this study has yielded valuable theoretical and practical insights, certain limitations remain that need to be addressed in future research.

At the research design level, this study employed a cross-sectional design. Although combining SEM and fsQCA revealed the predictive relationships and configurational paths among the variables, cross-sectional data cannot fully establish the dynamic temporal evolutionary relationships among the core variables. Future research should consider introducing longitudinal tracking designs or cross-lagged models to conduct multi-timepoint data collection on the same cohort, aiming to further verify the intertemporal stability and time-lag effects of the psychological intervention mechanisms proposed in this study (Cole and Maxwell, 2003).

Regarding sample representativeness, there are certain limitations in the sample scope and data representativeness. The data in this study focused on CUBAL female basketball players (*N* = 143), a cohort characterized by specific age ranges and competitive-level attributes. The generalizability of the research conclusions to professional league cohorts (e.g., WCBA), male athletes, or non-interactive sports requires further cross-sample validation. Future research could further expand the sample size and explore the heterogeneous characteristics of configurations associated with high performance anxiety among athletes of different genders and across different sports categories (e.g., open-skill vs. closed-skill sports), thereby enhancing the external validity and universality of the theoretical model (Swann et al., 2015).

Concerning measurement methodology, the measurement of variables primarily relied on subjective self-report questionnaires from the participants. Although the reliability and validity of the measurement models were confirmed during the data processing stage, self-assessments regarding “performance anxiety” and “fear of negative evaluation” in high-pressure competitive situations may still be subject to the potential influence of social desirability bias (Podsakoff et al., 2012). Future research could attempt to integrate multimodal measurement approaches, such as incorporating physiological and biochemical indicators, or combining objective behavioral evaluation data from a coach’s perspective, to cross-validate the formation mechanisms of performance anxiety from more objective physical and biological dimensions.

## Data Availability

The raw data supporting the conclusions of this article will be made available by the authors, without undue reservation.
